# Coix seed oil alleviates synovial angiogenesis through suppressing HIF-1α/VEGF-A signaling pathways via SIRT1 in collagen-induced arthritis rats

**DOI:** 10.1186/s13020-023-00833-6

**Published:** 2023-09-15

**Authors:** Qiangqiang Xu, Hongxi Kong, Shuang Ren, Fanyan Meng, Ruoshi Liu, Hongxin Jin, Jie Zhang

**Affiliations:** 1 Department of Chinese Medicine, The First Hospital of China Medical University, Liaoning, 110001 China; 2 Guangzhou University of Traditional Chinese Medicine, Guangdong, 510006 China

**Keywords:** Rheumatoid arthritis, Angiogenesis, SIRT1, HIF-1α, Coix seed oil

## Abstract

**Background:**

Rheumatoid arthritis (RA) is a chronic autoimmune disorder characterized by symmetric arthritis. Coix Seed Oil (CSO) has been shown to reduce inflammation in collagen induced arthritis (CIA) rats. However, the effect of CSO on synovial angiogenesis in RA is unknown. In this study, we aimed to explore whether CSO could inhibit RA synovial angiogenesis and elucidate the underlying mechanisms.

**Methods:**

CIA rat models were established and subjected to different doses of CSO treatments for four weeks in vivo. Arthritis index, paw swelling, and weight were recorded to assess clinical symptoms. Hematoxylin and Eosin staining, Safarnin O fast green staining, Micro-CT, Immunohistochemical, and Immunofluorescence (IF) staining were performed to examined changes in synovial and joint tissues. The serum HIF-1α and VEGF-A levels were evaluated through enzyme-linked immunosorbent assay. Fibroblast-like synoviocytes (FLS) of rats was stimulated with tumor necrosis factor-α (TNF-α) for developing inflammatory model in vitro. Optimal concentrations of CSO and TNF-α for stimulation were measured through Cell Counting Kit-8 test. Wound healing and Transwell migration experiments were employed to determine FLS migratory ability. IF staining was performed to assess HIF-1α nuclear translocation in FLS. Protein levels of SIRT1, HIF-1α, VEGF-A, and CD31 were assessed through Western blot. The isolated aortic rings were induced with recombinant rat VEGF-A 165 (VEGF-A_165_) to observe the CSO inhibitory impact on angiogenesis ex vivo.

**Results:**

CSO attenuated the progression of arthritis in CIA rats, mitigated histopathological deterioration in synovial and joint tissues, significantly inhibited immature vessels labeled with CD31^+^/αSMA^−^, and reduced the micro-vessels in VEGF-A_165_ induced aortic rings. Moreover, it upregulated SIRT1 protein levels in CIA rats and TNF-α induced FLS, but decreased HIF-1α and VEGF-A protein levels. Furthermore, CSO inhibited the migration ability and HIF-1α nuclear translocation of TNF-α induced FLS. Finally, suppressing SIRT1 levels in TNF-α induced FLS enhanced their migration ability, HIF-1α nuclear translocation, and the protein levels of HIF-1α, VEGF-A, and CD31, whereas the inhibitory effect of CSO on TNF-α induced FLS was severely constrained.

**Conclusions:**

This study indicates that CSO can alleviate synovial angiogenesis through suppressing HIF-1α/VEGF-A signaling pathways via SIRT1 in CIA rats.

**Supplementary Information:**

The online version contains supplementary material available at 10.1186/s13020-023-00833-6.

## Background

Rheumatoid arthritis (RA) is a chronic autoimmune disorder that causes symmetric arthritis [1]. The pathogenesis and pathophysiology of RA is still unknown. Nonetheless, angiogenesis is recognized as a crucial early event in RA progression. The immune response triggered by cytokine and chemokine causes inflammation of the synovium during the initial stages. This inflammation prompts the release of pro-angiogenesis factors such as Vascular Endothelial Growth Factor-A (VEGF-A), Fibroblast Growth Factor, and Angiopoietins [2]. Several studies have demonstrated a strong correlation between elevated VEGF-A levels and RA disease activity [3, 4]. VEGF-A can be secreted by Fibroblast-like synoviocytes (FLS) and specifically bind to its homologous receptors on Endothelial Cells (ECs). This promotes the proliferation and migration of ECs. Meanwhile, the permeability of blood vessels, which is necessary for new blood vessels to invade the inflamed synovium can be augmented by VEGF-A [5]. The newly formed blood vessels supply nutrients to the inflamed synovium, facilitate the infiltration of leukocytes, contribute to pannus formation, and ultimately result in cartilage erosion and joint destruction. Collectively, these processes induce structural damage in advanced-stage RA [6]. Thus, inhibition of angiogenesis may be an effective therapeutic strategy for RA that directly targets disease procession as opposed just suppressing the symptoms.

Hypoxia-inducible factor-1alpha (HIF-1α) plays an essential role in regulating cellular responses to hypoxia [7]. The hypoxic microenvironment, induced by the FLS proliferation, impedes HIF-1α breakdown and enhances its nuclear translocation, causing the transcription of VEGF-A target genes [8]. Sirtuin 1 (SIRT1) is a protein deacetylation-dependent kinase. Its transcriptional regulation is associated with several metabolic pathways, including nutrient deprivation, DNA damage, and oxidative stress [9]. The SIRT1 expression decreased in synovium and FLS of RA [10]. Previous studies have reported that synovial angiogenesis in collagen-induced arthritis (CIA) rats may be alleviated through the SIRT1/HIF-1α pathway [11, 12]. Therefore, the SIRT1-mediated HIF-1α/VEGF-A pathway may be an essential regulatory mechanism associated with RA angiogenesis.

Most modern conventional antirheumatic medications such as nonsteroidal anti-Inflammatory drugs and disease-modifying antirheumatic drugs, do not target angiogenesis directly as their primary mechanism of action. Some biological inhibitors such as infliximab and cyclosporin have been shown to reduce RA angiogenesis by inhibiting VEGF-A expression [13]. However, long-term administration can cause gastrointestinal distress and bone marrow suppression. Traditional Chinese Medicine (TCM) has gained popularity in recent years due its multi-target, good therapeutic effect, and few adverse effects. Coix Seed Oil (CSO), derived from the fruit of Chinese medicine Coix Seed, has been extensively investigated for its pharmacological properties, which include anti-inflammatory, anti-tumor, and improved glucolipid metabolism [14–16]. Previous research has shown that CSO decreases HIF-1α mRNA expression in Lewis lung cancer. CSO can mitigate oxidative stress and suppress the release of pro-inflammatory cytokines in CIA rats [17]. Moreover, we previously have demonstrated that CSO inhibited CIA rats from developing joint inflammation via the NLRP3/caspase-1 pathway [data not shown]. However, it remains uncertain whether CSO can inhibit synovial angiogenesis and modulate SIRT1 expression. In this study, we evaluated the effect of CSO on synovial angiogenesis by constructing CIA rat models in vivo and aortic rings induced with recombinant rat VEGF-A 165 (VEGF-A_165_) ex vivo. Additionally, we inhibited SIRT1 expression in vitro to investigate the relationship between SIRT1 and HIF-1α, and to clarify whether SIRT1 is the direct target of CSO in alleviating synovial angiogenesis.

## Methods and materials

### Drugs and reagents

CSO was procured through Zhejiang Kanglaite Pharmaceutical CO., Ltd. (Zhejiang, China). Methotrexate (MTX) was purchased from Shanghai Pharma CO., Ltd. (Shanghai, China). Chick type II collagen and complete Freund’s adjuvant (CFA) were obtained from Chondrex (Washington, USA). Enzyme-linked immunosorbent assay (ELISA) kits for HIF-1α and VEGF-A were purchased from Shanghai Guido Biotechnology (Shanghai, China). Phenyl Methane Sulfonyl Fluoride (PMSF), Radio Immunoprecipitation Assay (RIPA) lysis buffer, 4’,6-diamidino-2-phenylindole (DAPI), Annexin V-FITC/Propidium Iodide (PI) apoptosis kit, and Bicinchonincacid (BCA) protein detection kit were purchased from Beyotime Biotechnology (Shanghai, China). Diaminobenzidine tetrahydrochloride (DAB) and hematoxylin were purchased from MXB Biotechnologies (Beijing, China). Safranin O fast green Kit was purchased from Solarbio Life Scinces (Beijing, China). TGX Stain-Free™ Acrylamide Kit and protein marker were purchased from Bio-Rad (California, USA). Anti-SIRT1 and Anti-Vimentin antibody were purchased from Abcam (Cambridge, UK). Anti-HIF-1α, Anti-VEGF-A, Anti-CD31, Anti-αSMA, Anti-GAPDH, Goat Anti-Rabbit IgG (H + L) HRP, Goat Anti-Mouse IgG (H + L) HRP, Goat Anti-Rabbit IgG (H + L) CY3-conjugated, and Goat Anti-Mouse IgG (H + L) FITC-conjugated antibody were procured through Affinity (Jiangsu, China). Fetal bovine serum (FBS) was obtained from Clark bioscience (Virginia, USA). High glucose Dulbecco’s modified Eagle’s medium (H-DMEM), Penicillin-Streptomycin Solution, phosphate-buffered saline (PBS), together with 0.25% Trypsin-EDTA were procured through Thermo Fisher (Massachusetts, USA). Recombinant rat TNF-α, Recombinant rat VEGF-A 165 (VEGF-A_165_) and Transwell chambers were purchased from PeproTech (New Jersey, USA). The Matrigel was purchased from Corning (New York, USA). EX527 (SIRT1 inhibitor) was acquired from MedChemExpress (New Jersey, USA).

### Experimental animals

Six-week-old male Sprague-Dawley rats (200 ± 20 g) were procured through Beijing HFK Bioscience Co., Ltd. and housed in the Department of Laboratory Animal Science under specific pathogen-free (SPF) conditions (Humidity 40-60%; 12/12 h day/night cycle; temperature 24 ± 2 °C).

### Establishment of CIA models and drug administration

The CIA rat models were established based on the protocol described previously [18]. Briefly, 0.1 M acetic acid was used to dissolve Chick type II collagen, and this solution was emulsified with an equivalent proportion of CFA. The rats were immunized through intradermal injections at the tail base, followed by a second immunization after 7 days. Rats were divided randomly into six groups: normal control group (CON, n = 6), CIA model group (CIA, n = 6), CSO low-dose group (CSO-L, 2.1 g/kg/day, n = 6), CSO medium-dose group (CSO-M, 4.2 g/kg/day, n = 6), CSO high-dose group (CSO-H, 8.4 g/kg/day, n = 6), and MTX group (MTX, 0.5 mg/kg, 3 times/week. n = 6). Equal amounts of 0.9% saline were administered to all rats in the CON and CIA groups. Following the secondary immunization, the rats in each group received continuous treatment for a duration of four weeks. The day of first immunization was designated as day 0, and medication treatments were administered from day 14 to 35.

### Assessment of arthritis

The arthritis index of rats was scored (ranging from 0 to 4) every 3 days post-the first immunization (day 0). The specific details of the AI scoring are as follows [19]: (0) without swelling; (1) toe joint having slight swelling; (2) mild swelling extending from hindfoot to ankle; (3) moderate swelling or even erythema of ankle; (4) extreme erythema and swelling, even an ankle-to-toe joint ulcer, and bleeding. Moreover, the width of the rat’s right hind ankle joint was measured using vernier calipers, and the rats’ weights were recorded on a weekly basis.

### Histological analysis

The ankle joint and synovial tissue of the knee joint were fixed in 4% paraformaldehyde (PFA), paraffinized, and cut into 4-µm-thick sections. Histopathological changes in the joints were evaluated through Hematoxylin and Eosin (H&E) staining. As previously reported [20], the joint pathology score was graded on a scale of 0**–**4 (0: normal joint synovium and bone; 1: FLS proliferation and inflammatory cell infiltration; 2: pannus formation and cartilage erosion; 3: extensive cartilage and bone damage; 4: joint adhesions and disability). Furthermore, the safranin O fast green kit was employed to assess cartilage erosion in the joints. The cartilage erosion score ranged from 0 to 4 (0: no cartilage erosion; 1: minimal erosion less than 10% of the articular cartilage; 2: erosion up to 30% of the articular cartilage; 3: erosion up to 50% of the articular cartilage; 4: severe erosion over 50% of the articular cartilage) [21]. Finally, as described previously [22]. H&E staining was utilized to observe the vessel density of knee synovial tissues, quantified by counting the number of vessel-like structures per unit area of synovial tissue.

### Micro-CT

According to previous studies [23–25]. the ankle joints were fixed with 4% PFA for 48 h and subsequently scanned using SkyScan1276 Micro-CT (Bruker, Kontich, Belgium) with the following parameters: Source Voltage of 70 kV, Source Current of 200 µA, Exposure time of 388ms, and no 360-degree rotation. Three-dimensional (3D) reconstruction of the two-dimensional joint scan images was performed using Recon software (Bruker, Kontich, Belgium). A cuboid area in the right calcaneus (size: 2.0268 × 1.0134 × 1.0134 mm^3^) was selected as the Region of Interest (ROI) for analyzing trabecular bone changes. The percent bone volume (BV/TV), bone mineral density (BMD), trabecular number (Tb.N), Trabecular thickness (Tb.Th), Trabecular separation (Tb.Sp), and Trabecular pattern factor (Tb.Pf) were measured using CTAn software (Bruker, Kontich, Belgium). Each parameter was measured at least three times.

### ELISA analysis

The serum protein levels of HIF-1α and VEGF-A in rats were quantified using corresponding ELISA kits, following the instructions provided by the kits.

### Immunohistochemical (IHC) assessment

Initially, the rat synovial tissues were treated with Anti-CD31 primary antibody (1:150 dilution; overnight/4 °C), and then with Goat Anti-Rabbit IgG (H + L) HRP-conjugated secondary antibody (1:200 dilution) at room temperature (RT) for 2 h. Subsequently, DAB was used for color development and hematoxylin for nuclear staining. Eventually, a semi-quantitative analysis was conducted as described previously [26].

### Immunofluorescence (IF) analysis

Synovial tissues were incubated with Anti-CD31 (1:150 dilution) and Anti-αSMA (1:200 dilution) primary antibodies, followed by Goat Anti-Rabbit IgG (H + L) CY3-conjugated secondary antibody (1:200 dilution) and Goat Anti-Mouse IgG (H + L) FITC-conjugated secondary antibody (1:200 dilution), and stained with DAPI. In addition, FLS was fixed with 4% PFA for 30 min, permeabilized with 0.5% Triton-X 100 for 30 min, and incubated with 5% BSA for 1 h to block non-specific binding. The blocked FLS were incubated with Anti-Vimentin antibody (1:250 dilution) or Anti-HIF-1α primary antibody (1:100 dilution) overnight at 4 ℃, and then incubated for 2 h at RT with goat anti-mouse IgG (H + L) FITC-conjugated secondary antibody (1:200 or 250 dilution).

### Cell culture and treatment

According to the study reported previously [27], rat primary Fibroblast-like synoviocytes (FLS) were isolated as follows: six-week-old male Sprague-Dawley rats were euthanized, and the synovial tissues were extracted under sterile conditions. Synovial tissues were carefully cut into small pieces of roughly 1**–**1.5 mm^3^ post-the removal of fatty and fibrous tissues. Subsequently, the pieces were evenly distributed on the bottom of 50 ml cell culture flasks. The flasks were then placed vertically in a CO_2_ incubator (5% CO_2_, 37 °C) for 4 h to allow adhesion of the tissue pieces onto the flask surface. Next, the flasks were returned to a horizontal position, and the FLS culture medium comprising H-DMEM supplemented with 20% FBS, 100 units/ml penicillin, and 100 µg/ml streptomycin was added. The synovial tissues were removed once a sufficient number of FLS crawled out from the tissue periphery. Finally, the FLS were cultivated and allowed to proliferate for 3 to 6 generations.

FLS were separated into five groups, each receiving a different treatment: Negative Control group (NC, n = 3), TNF-α group (TNF-α, n = 3), TNF-α + CSO group (TNF-α + CSO, n = 3), TNF-α + EX527 group (TNF-α + EX527, n = 3), and TNF-α + EX527 + CSO group (TNF-α + EX527 + CSO, n = 3).

### CCK8 assay

FLS (5 × 10^3^ cells/well) were seeded into each well of 96-well plates. CSO or TNF-α (100 µl/well of different concentrations) was added to the wells and incubated for 24 or 48 h. The CSO concentrations were as follows: 0, 12.5, 25, 50, 100, 200, 400, 800, 1000, 2000, and 4000 µg/ml. The TNF-α concentrations were as follows: 0, 2.5, 5, 10, and 20 ng/ml. After rinsing with PBS, FLS were cultured in 100 µl of H-DMEM supplemented with CCK8 solution (10 µl) for 2 h. The absorbance of FLS (450 nm) was analyzed using an enzyme marker.

### Cell apoptosis assay

FLS (1 × 10^5^ cells/well) were plated in 6-well plates and treated with CSO for 48 h. Following the apoptosis kit protocol, FLS were suspended in 500 µl of 1 × Binding Buffer, then incubated with 5 µl of Annexin V-FITC and 10 µl of PI for 5 min at RT. The percentage of apoptosis cells were measured using FACSCalibur flow cytometer (San Jose, California, USA) and the data were analyzed by FlowJo software (San Jose, California, USA).

### Wound healing assay

In 6-well plates, FLS (1 × 10^5^ cells/well) were placed into incubation for 24 h. Scratch lines were created on the plates using 200 µl pipette tips. Each well was placed with 2 ml of the corresponding treatment reagent for 48 h. The cell-covered area was calculated using ImageJ software to indicate the wound-healing ability of FLS.

### Transwell migration assay

The Transwell chamber with an 8 μm hole size membrane was used to perform the Transwell migration assay. Briefly, 300 µl FLS (5 × 10^4^ cells/ml) solution, containing CSO or EX527 or CSO + EX527, were added into the upper chambers; The lower chambers were coated with a chemoattractant consisting of a 600 µl solution of 20% FBS HDMEM, including TNF-α. The migrating cells were fixed and stained with a crystal violet solution after 20 h. The relative number migrated cells was calculated using ImageJ software.

### Western blot analysis

The synovial tissues of rats or FLS were lysed in RIPA lysis solution comprising 1% PMSF. The BCA protein detection kit was utilized to quantify the protein concentration. Protein lysate (30 µg) was separated on 10-12% SDS-PAGE gel (120 V, 90-120 min) and subsequently transferred onto PVDF membrane (220 mA, 1 KD/min). The membrane was incubated with the corresponding primary antibodies overnight at 4 °C after blocking with 5% BSA solution at RT for 30 min. The membrane was washed with TBST and incubated with HRP-conjugated secondary antibodies (RT, 120 min). GAPDH (1:3000 dilution) served as the internal control. Western blot analysis was performed using the primary antibodies: Anti-SIRT1 (1:1000 dilution), Anti-HIF-1α (1:1000 dilution), Anti-VEGF-A (1:1000 dilution), and Anti-CD31 (1:1000 dilution) respectively. The secondary antibodies were: Goat Anti-Rabbit IgG (H + L) HRP-conjugated secondary antibodies (1:3000 dilution) and Goat Anti-Mouse IgG (H + L) HRP-conjugated secondary antibodies (1:3000 dilution).

### Rat aortic rings assay

A 48-well plate was pre-coated with matrigel and incubated at 37 °C with 5% CO_2_ for 30 min to allow the matrigel to solidify on the plates. The rat aortas were isolated and sectioned into 1**-**1.5 mm rings. These rings were subsequently placed in the pre-coated 48-well plates. The wells were supplemented with a mixture of 10% FBS, 90% H-DMEM, 20 µg/l VEGF-A_165_, and CSO. Post-7 days, the status of angiogenesis within the rat thoracic aortic rings was observed.

### Statistical analysis

GraphPad Prism 8.4.2 software (San Diego, USA) was employed for analyzing the data of this study. The datasets were presented as the mean ± standard deviation (SD). Kruskal-Wallis tests was applied to analysis non-parametric data. The Student’s t test was used to compare differences between the two groups. One-way analysis of variance (ANOVA) with Tukey’s post-hoc test was utilized to compare differences among groups. *P* < 0.05 or *P* < 0.01 was set as the threshold of the statistical significance.

## Results

### CSO alleviated the clinical symptoms of CIA rats

**Fig. 1:**
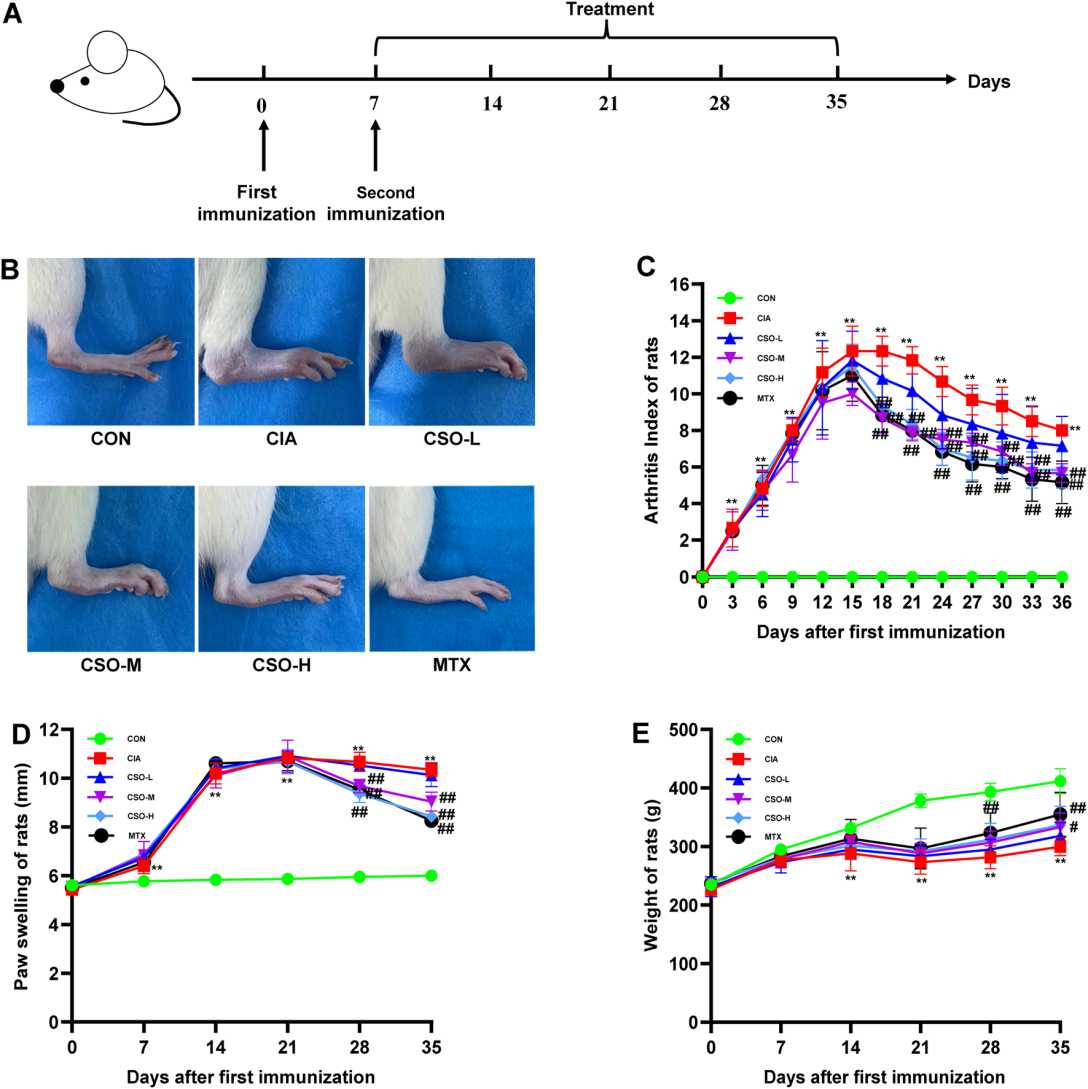
CSO alleviated the clinical symptoms of CIA rats. **A** An illustration of the Experimental procedure. **B** Representative picture for ankle joints. **C** Arthritis index of rats. **D** Paw swelling of rats (mm). **E** Weight of rats (g). Datasets are shown as the mean ± SD. *P < 0.05, **P < 0.01 vs. CON group; ^#^*P* < 0.05, ^##^*P* < 0.01 vs. CIA group, n = 6

The CIA models were established to evaluate the in vivo effect of CSO on arthritis. Figure 1A displayed the experimental procedure. The results shown in Fig. 1B-E demonstrated a significant increase in the arthritis index and paw swelling in CIA rats on day 7 following the first immunization. However, after four weeks of administration of CSO-M and CSO-H, there was a notable reduction in the arthritis index and paw swelling. Additionally, the weight of rats was also utilized as an indirect indicator to evaluate arthritis progression. From day 14 onwards, the weight of CIA rats was appreciably lower than that of the CON rats, reaching its lowest point on day 21 before gradually recovering. Notably, the CSO-H group exhibited a significantly higher weight in comparison to the CIA group on day 35. These findings indicated that high-dose CSO could effectively alleviate the progression of arthritis in CIA rats.

### CSO improved histopathological deteriorations of CIA rats

**Fig. 2:**
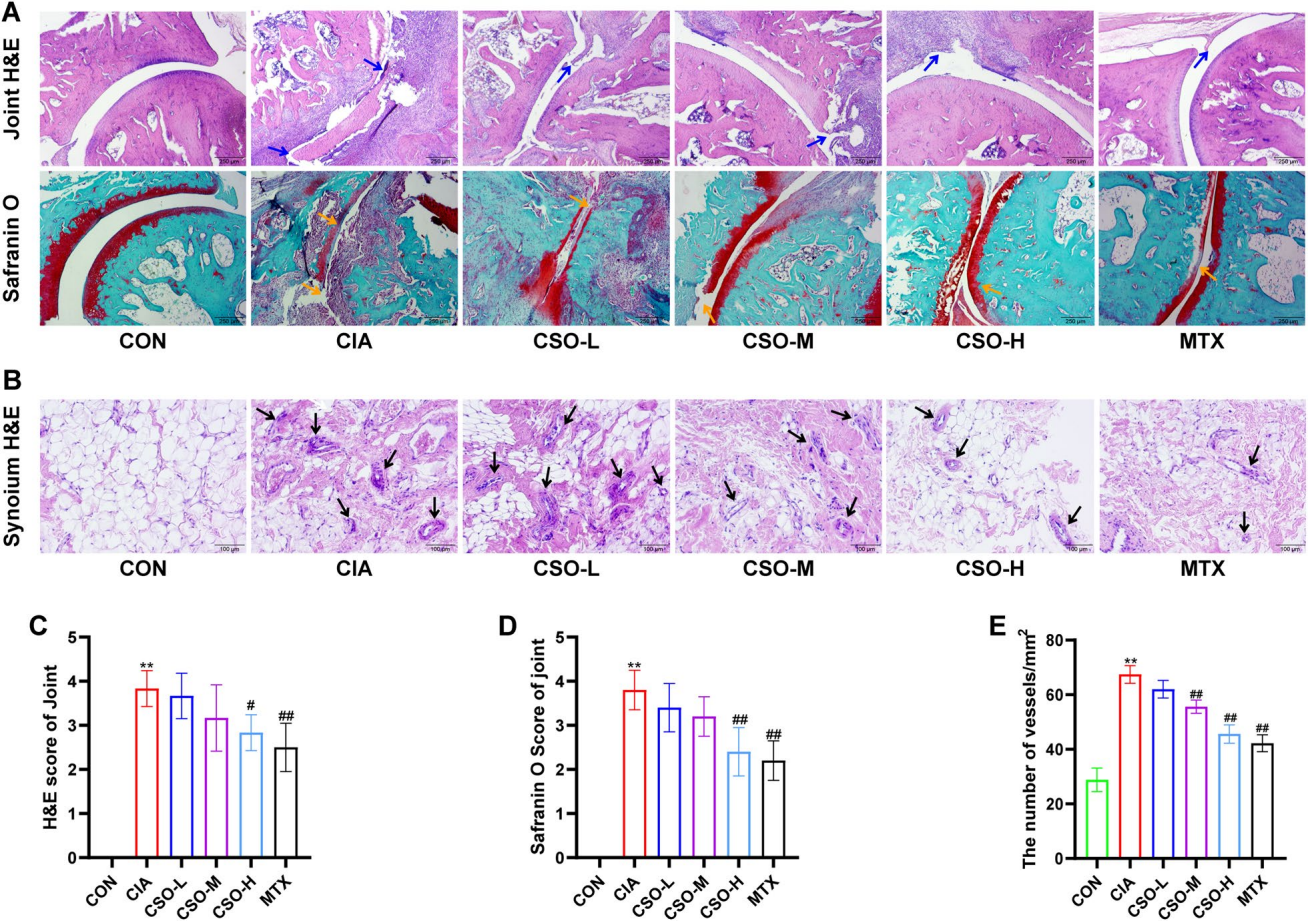
CSO improved histopathological deteriorations in the joint and synovium of CIA rats. **A** H&E staining and Safranin O fast green staining of ankle joints. Blue arrows indicate inflammatory infiltration. Yellow arrows indicate cartilage erosion. **B** H&E staining of synovial tissues. Black arrows indicate vessel-like structures. **C** H&E score of joint. **D** Safranin O score of joint. **E** The number of vessel-like structures in synovial tissues. Datasets are shown as the mean ± SD. *P < 0.05, **P < 0.01 vs. CON group; ^#^*P* < 0.05, ^##^*P* < 0.01 vs. CIA group, n = 6

**Fig. 3:**
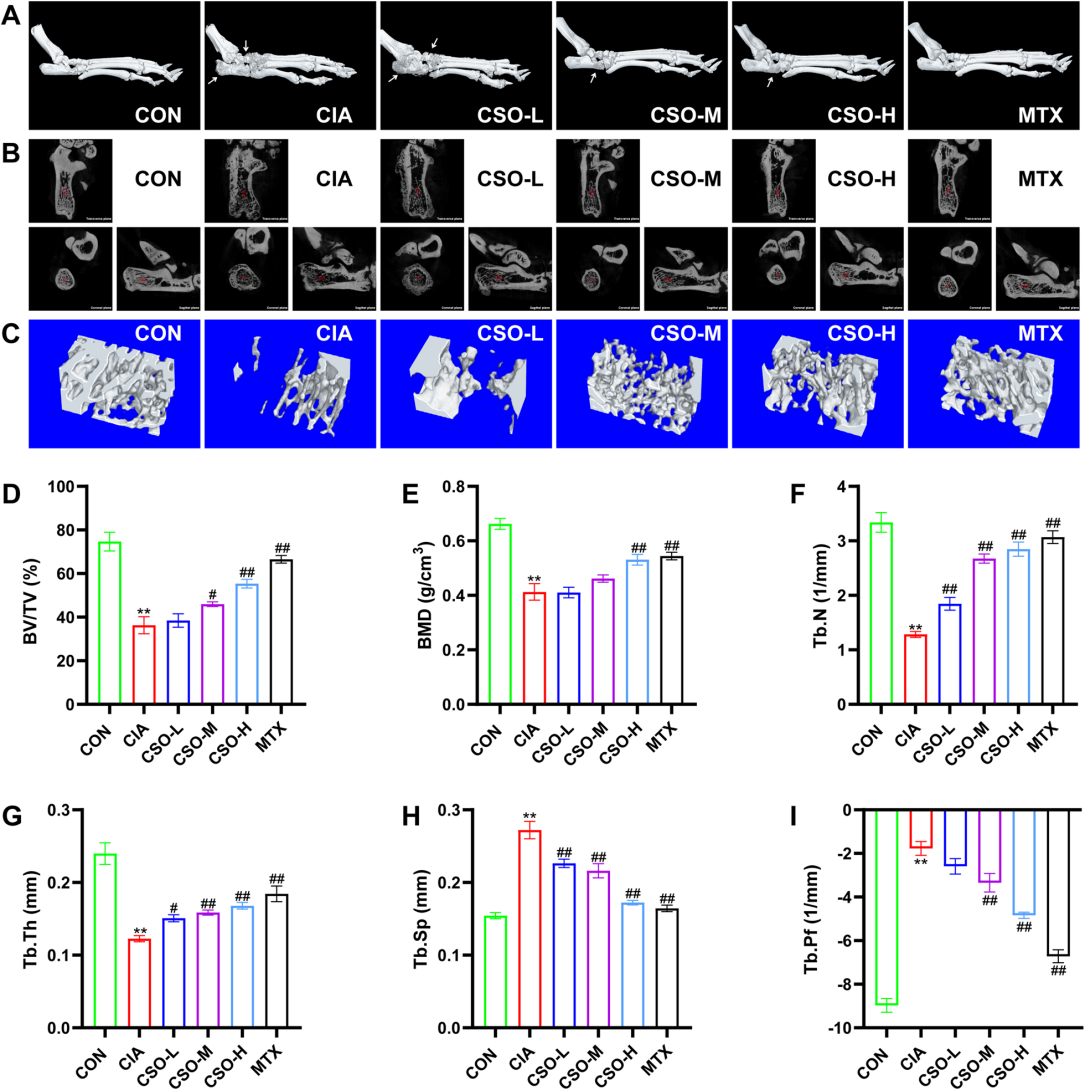
CSO reduced joint destruction of CIA rats. **A** Representative micro-CT pictures of the ankle joints. White arrows indicate joint destruction. **B** Representative micro-CT scans of the calcaneus in three planes (Transverse, Coronal, and Sagittal). The red area designates the ROI in the calcaneus. **C** Representative 3D reconstruction pictures of the ROI. **D** BV/TV (%). **E** BMD (g/cm^3^). **F** Tb.N (1/mm). **G** Tb.Th (mm). **H** Tb.Sp (mm). **I** Tb.Pf (1/mm). Datasets are shown as the mean ± SD. *P < 0.05, **P < 0.01 vs. CON group; ^#^*P* < 0.05, ^##^*P* < 0.01 vs. CIA group, n = 6

We subsequently evaluated the effect of CSO on the pathology of joints and synovial tissues in CIA rats. As shown in Figs. 2 and 3, CSO-H substantially mitigated H&E score of joint. Meanwhile, Safranin O fast green staining and Micro-CT were employed to investigate the pathological changes following angiogenesis. High-dose CSO considerably decreased the Safaranin O cartilage score and mitigated joint destruction. Furthermore, the analysis of bone parameters in the calcaneus ROI showed significant improvements in BV/TV, BMD, Tb.N, and Tb.Th in the CSO-H group compared to the CIA group. Conversely, there was a notable reduction in Tb.Sp and Tb.Pf. Additionally, we examined the knee synovial tissue using H&E staining. The number of vascular-like structures within the CSO-M and CSO-H groups were significantly reduced, suggesting the potential role of CSO in inhibiting vessel formation. These results showed that high-dose CSO improved histopathological deterioration in joints and synovial tissues in CIA rats.

### CSO reduced the serum levels of HIF-1α and VEGF-A in CIA rats

**Fig. 4:**
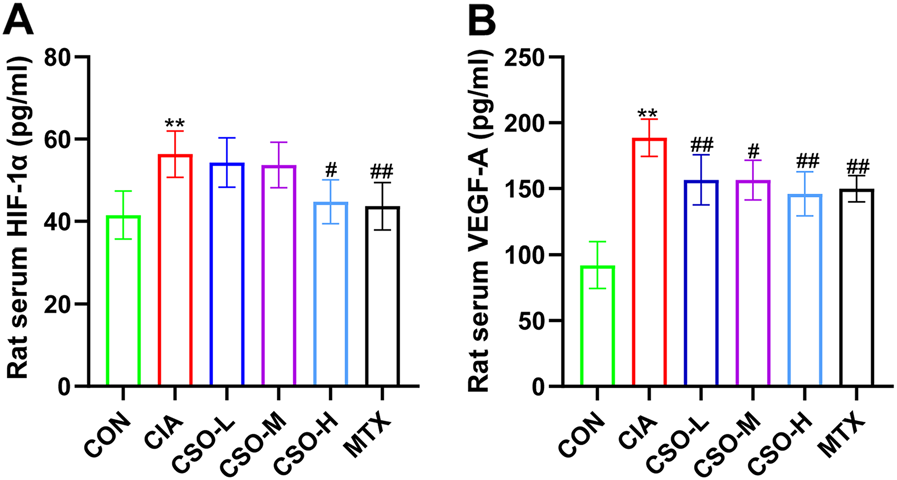
CSO reduced the serum levels of HIF-1α and VEGF-A in CIA rats. **A** Serum level of HIF-1α in rats. **B** Serum level of VEGF-A in rats. Datasets are shown as the mean ± SD. *P < 0.05, **P < 0.01 vs. CON group; ^#^*P* < 0.05, ^##^*P* < 0.01 vs. CIA group, n = 6

The ELISA kits were used to quantify serum HIF-1α and VEGF-A levels. Figure 4 showed the elevated levels of HIF-1α and VEGF-A in the CIA group compared to the CON group. While the CSO-L and CSO-M groups showed a slight, non-significant decrease in HIF-1α levels compared to the CIA group. Notably, the CSO-H group had considerably lower HIF-1α levels than the CIA group, indicating the effective suppression of HIF-1α elevation in CIA rats by high-dose CSO. In Addition, the CSO-H group displayed the most pronounced reduction in serum VEGF-A levels differential from the CIA group, suggesting that high-dose CSO could substantially decrease VEGF-A serum levels in CIA rats.

### CSO inhibited angiogenesis in synovial tissues of CIA rats

**Fig. 5:**
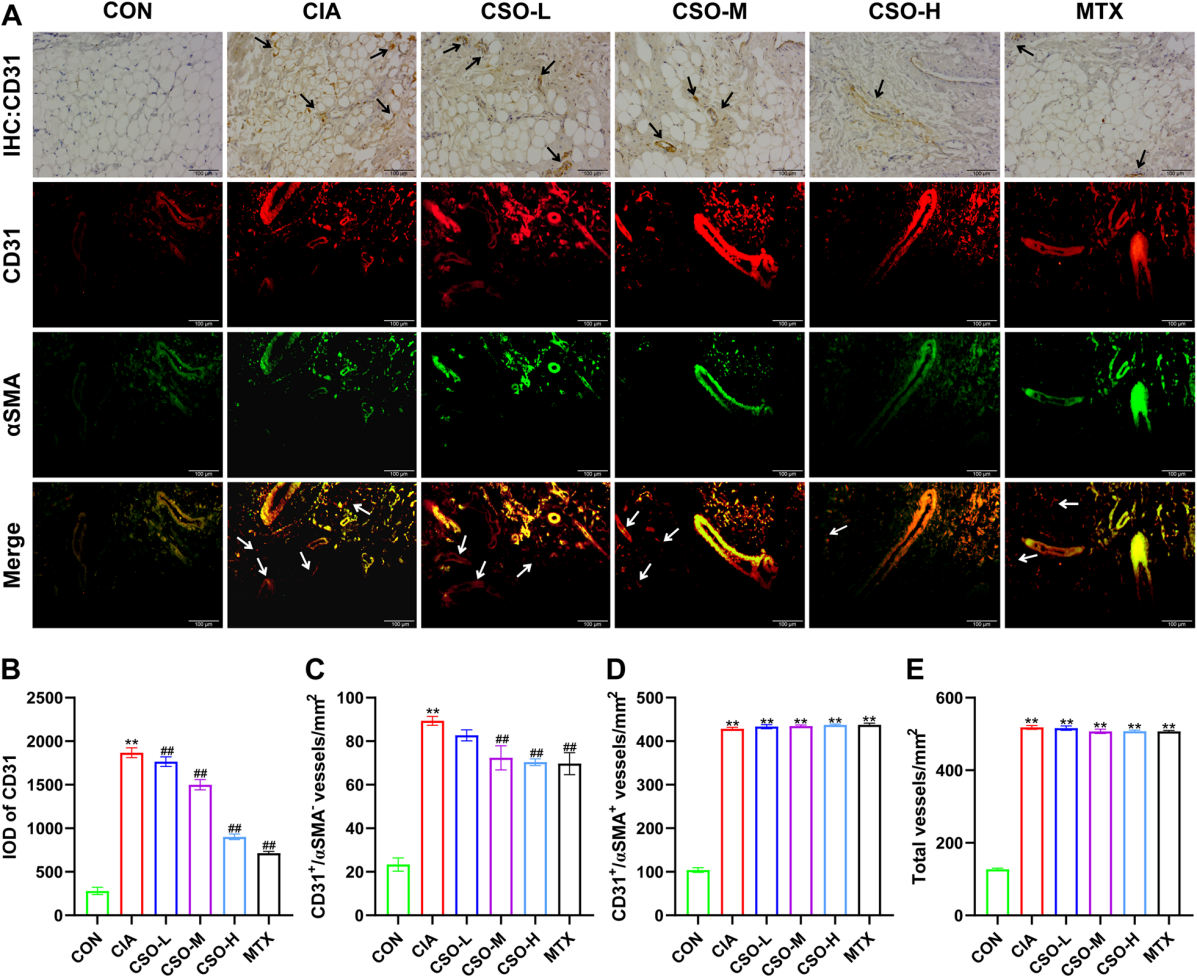
CSO inhibited angiogenesis in synovial tissues of CIA rats. **A** IHC staining of CD31 and IF staining of CD31/αSMA. Black arrows indicate positive staining of CD31, White arrows indicate immature vessels labeled with CD31^+^/αSMA^−^. **B** Integral Optical Density (IOD) of CD31. **C** The number of CD31^+^/αSMA^−^ vessels. **D** The number of CD31^+^/αSMA^+^ vessels. **E** The number of Total vessels. Datasets are shown as the mean ± SD. *P < 0.05, **P < 0.01 vs. CON group; ^#^*P* < 0.05, ^##^*P* < 0.01 vs. CIA group, n = 3

CD31, a prevalent marker to quantify angiogenesis [28], was evaluated through IHC and IF assays to assess the anti-angiogenesis potential of CSO in our research. According to Fig. 5, the level of CD31 was significantly higher in the CIA group than in the CON group. Conversely, the positive staining rate of CD31 in synovial tissues was evidently decreased within CSO treatment groups compared to the CIA group. The CIA group also exhibited a significantly higher number of immature vessels that were CD31^+^/αSMA^−^ labeled compared to the CON group. The CSO-M and CSO-H groups showed substantially low angiogenesis in comparison to the CIA group, indicating that CSO exerted a dose-dependent effect on anti-angiogenesis. No statistically significant variation was observed among the modeled groups, despite a considerable increase in the number of CD31^+^/αSMA^+^ labeled mature vessels and total vessels in all modeled groups compared to the CON group. Such datasets strongly suggested that CSO exerted a potent inhibitory effect on angiogenesis in CIA rats.

### **CSO regulated the expression levels of SIRT1, HIF-1α, VEGF-A, and CD31 in synovial tissues of CIA rats**

**Fig. 6:**
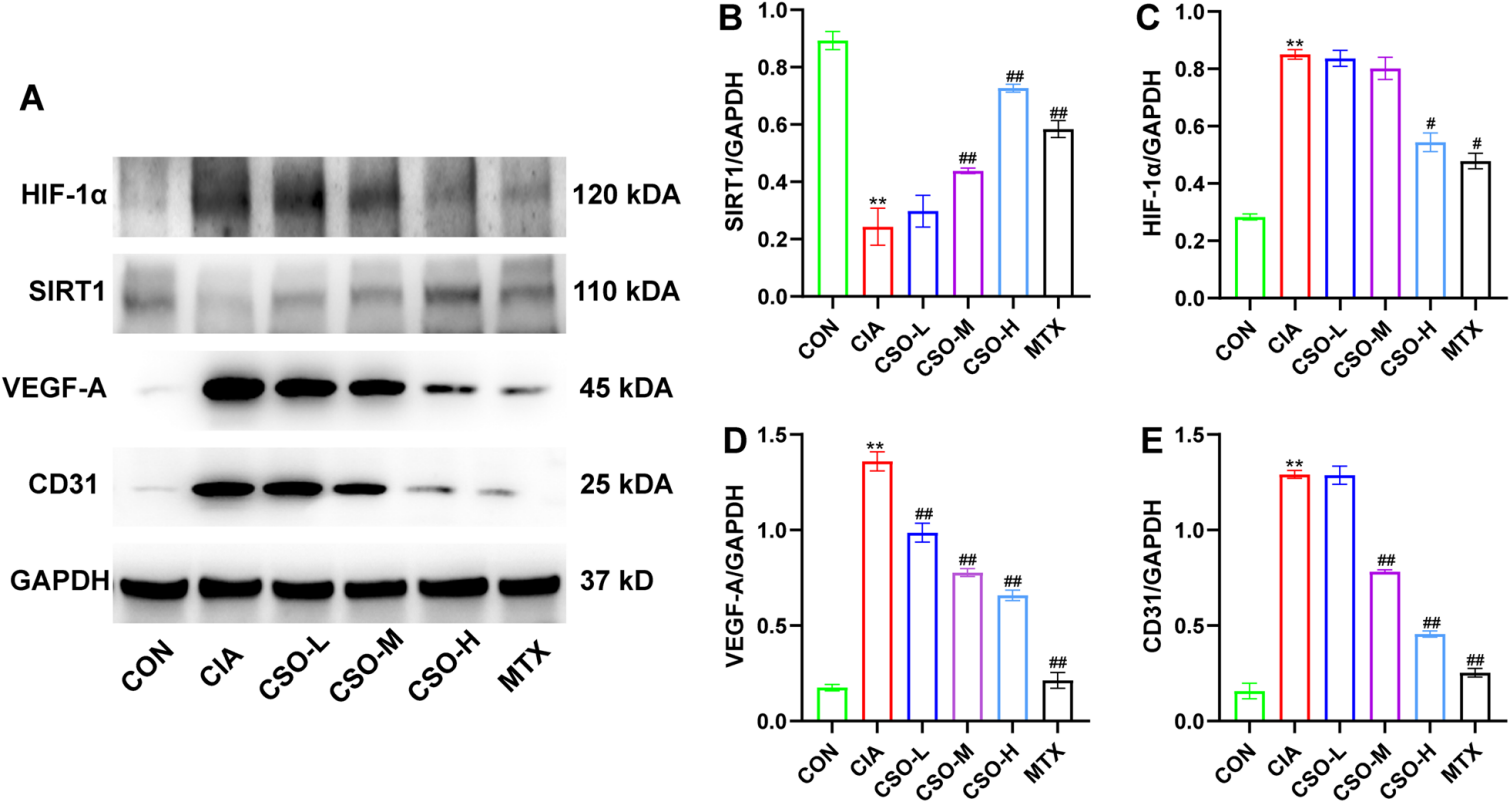
CSO regulated the expression levels of SIRT1, HIF-1α, VEGF-A, and CD31 in CIA rats. **A** Representative picture of Western blot. **B** SIRT1/GAPDH. **C** HIF-1α/GAPDH. **D** VEGF-A/GAPDH. **E** CD31/GAPDH. Datasets are shown as the mean ± SD. *P < 0.05, **P < 0.01 vs. CON group; ^#^*P* < 0.05, ^##^*P* < 0.01 vs. CIA group, n = 3

According to Fig. 6, the CIA group had considerably lower levels of SIRT1 than the CON group. Conversely, HIF-1α, VEGF-A, and CD31 expression levels were remarkably elevated in the CIA group. SIRT1 expression was not significantly different between CSO-L and CIA groups. however, it was significantly higher in the CSO-M, CSO-H, and MTX groups when compared to the CIA group. In contrast, HIF-1α expression was significantly reduced in the CSO-H and MTX groups. The CSO treatment and MTX groups displayed decreased VEGF-A expression in comparison to the CIA group, indicating a dose-dependent inhibitory effect of CSO on VEGF-A expression. CD31 expression in the CSO-L group did not differ from that of the CIA group. However, CD31 expression was significantly reduced in the CSO-M, CSO-H, and MTX groups compared to the CIA group. In summary, high-dose CSO increased SIRT1 levels and decreased HIF-1α, VEGF-A, and CD31 levels in CIA rats.

### Identification of primary FLS of rats

**Fig. 7:**
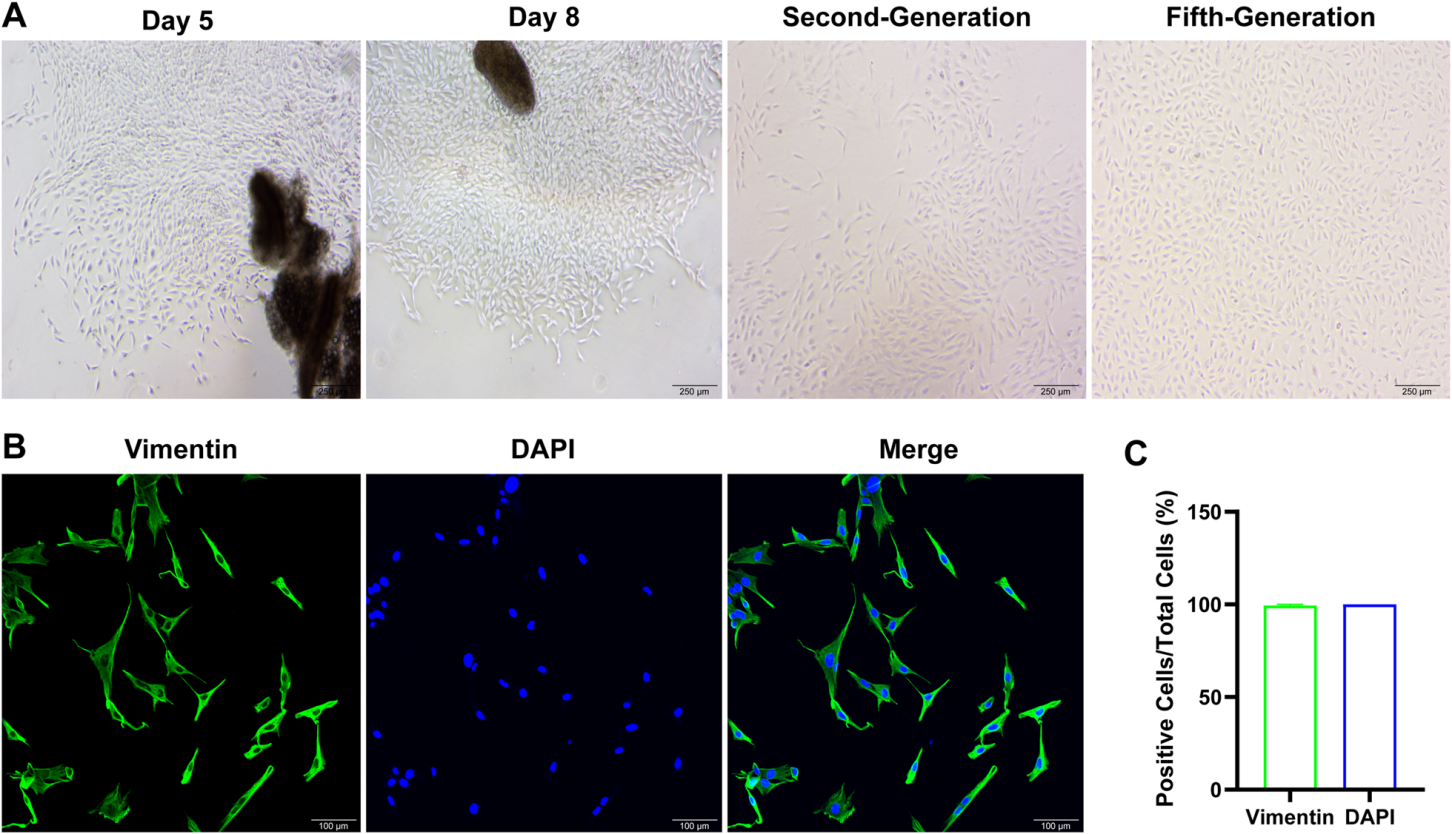
Identification of primary FLS of rats. **A** Representative picture of FLS at different days and generations. **B** Representative picture of Vimentin-positive fluorescent staining. **C** Vimentin positive rate. Datasets are shown as the mean ± SD. *P < 0.05, **P < 0.01 vs. DAPI, n = 3

Primary FLS of rats were isolated to investigate the underlying mechanism of CSO in treating angiogenesis. As shown in Fig. 7, FLS merged from the periphery of the synovial tissue and exhibited radial growth on day 5. From the second-generation, FLS exhibited a regular, elongated spindle-shaped morphology with centrally positioned oval nuclei. FLS is characterized by unique surface markers such as CD55, VCAM-1, and Vimentin, which distinguish them from other types of fibroblasts [29]. Therefore, we performed IF staining of third-generation FLS using Vimentin, and observed Vimentin positive rate exceeding 98.7%, confirming the identity of the cells as FLS.

### Effects of different concentrations of TNF-α on cell proliferation and SIRT1 expression

**Fig. 8:**
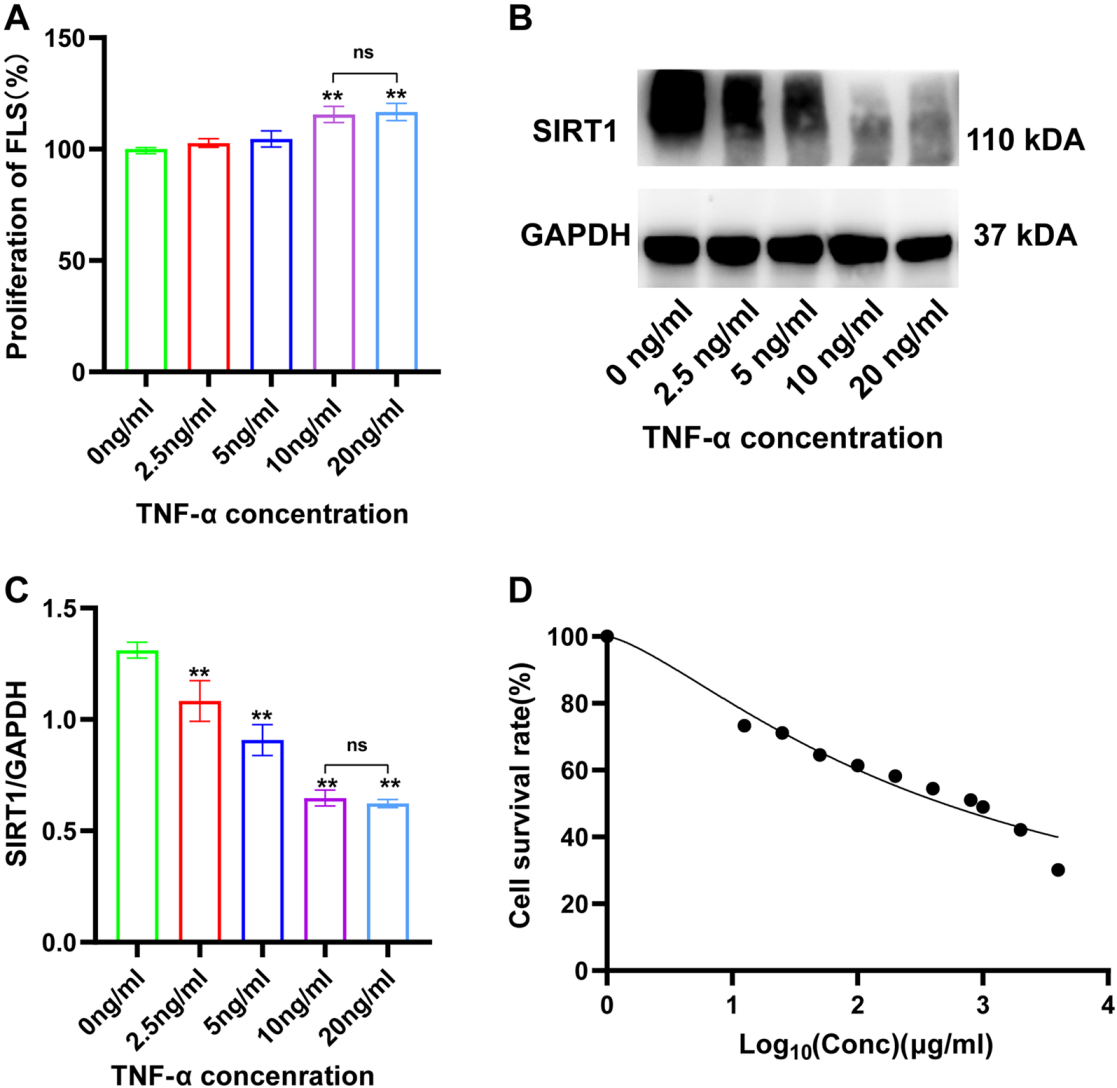
Effects of different concentrations of CSO and TNF-α on cell proliferation and SIRT1 expression. **A** FLS Proliferation rate (%). **B** Representative picture of SIRT1 levels. **C** SIRT1/GAPDH. **D** Cell survival rate (%). Datasets are shown as the mean ± SD. *P < 0.05, **P < 0.01 vs. 0 ng/ml TNF-α group, n = 3

FLS were stimulated with five different doses of TNF-α (0, 2.5, 5, 10, and 20 ng/ml) for 24 h. As demonstrated in Fig. 8A-C, 10 and 20 ng/ml TNF-α groups exhibited higher proliferation compared with the 0 ng/ml group. In contrast, SIRT1 expression was markedly reduced in 10 and 20 ng/ml groups compared to 0 ng/ml group. However, there was no discernible difference in proliferation rates and SIRT1 expression between these two groups. Intriguingly, while SIRT1 expression significantly declined in the 2.5 and 10 ng/ml groups, this did not correspond with the change in proliferation rates. Consequently, 10 ng/ml TNF-α was selected to establish inflammatory model *in vitro.*

### Effects of different CSO concentrations on the viability of FLS

As indicated in Fig. 8D, FLS were cultured with 11 different concentrations of CSO (0**–**4000 µg/ml) for 48 h. FLS viability consistently decreased with increasing CSO concentration. The estimated value for half maximum inhibitory concentration (IC50) of CSO on FLS was 625 µg/ml. Subsequently, to determine the optimal concentrations of CSO to treat TNF-α induced FLS, FLS were cultured with 6 different concentrations of CSO (0, 125, 250, 375, 500, and 625 µg/ml) and/or 10 ng/ml TNF-α. As shown in Additional file 1 and Additional file 2, the viability of TNF-α induced FLS exceeded 80% that of normal cells when treated with CSO concentrations below 500 µg/ml. Furthermore, the percentage of apoptosis cells in the CSO (500 µg/ml) group exhibited no significant difference in comparison to the NC group. Treating FLS with 10 ng/ml TNF-α could reduce apoptosis. Although there was a slight increase in the apoptosis rate in the TNF-α (10 ng/ml) + CSO (500 µg/ml) group, no significant difference was observed compared to the TNF-α group. Thus, CSO concentration of 500 µg/ml was chosen for subsequent experiments.

### CSO inhibited the migration ability of TNF-α induced FLS proliferation

**Fig. 9:**
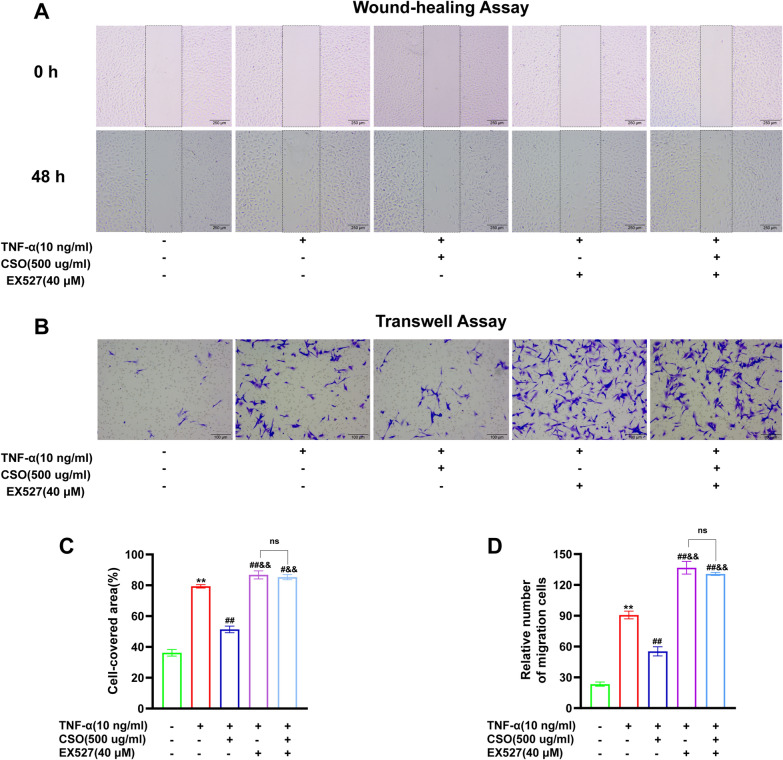
CSO inhibited the migration ability of TNF-α induced FLS proliferation. **A** Representative image of wound-healing assay. **B** Representative image of Transwell migration assay. **C** FLS-covered area (%). **D** Relative number of migration cells. Datasets are shown as the mean ± SD. *P < 0.05, **P < 0.01 vs. NC group; ^#^*P* < 0.05, ^##^*P* < 0.01 vs. TNF-α group; n = 3. ^&^*P* < 0.05, ^&&^*P* < 0.01 vs. TNF-α + CSO group, n = 3

Wound-healing and Transwell migration assays were performed to assess the effect of CSO on the horizontal and vertical migration abilities of TNF-α induced FLS. As shown in Fig. 9, The cell-covered area and the number of migrated FLS in the TNF-α group were dramatically decreased after 48 h of CSO treatment. The FLS migration was substantially enhanced in the TNF-α + EX527 group compared with the TNF-α group. Interestingly, unlike the TNF-α + CSO group, the enhanced migration was not suppressed in the TNF-α + EX527 + CSO group, and no discernible difference was observed between the TNF-α + EX527 + CSO and TNF-α + EX527 groups. These findings indicated that CSO suppressed FLS migration, but this effect was attenuated following inhibition of SIRT1 expression. It suggested that SIRT1 regulated FLS migration, and CSO might influence migration by modifying SIRT1.

### CSO modulated the expression levels of SIRT1, HIF-1α, VEGF-A, and CD31 in TNF-α induced FLS

**Fig. 10:**
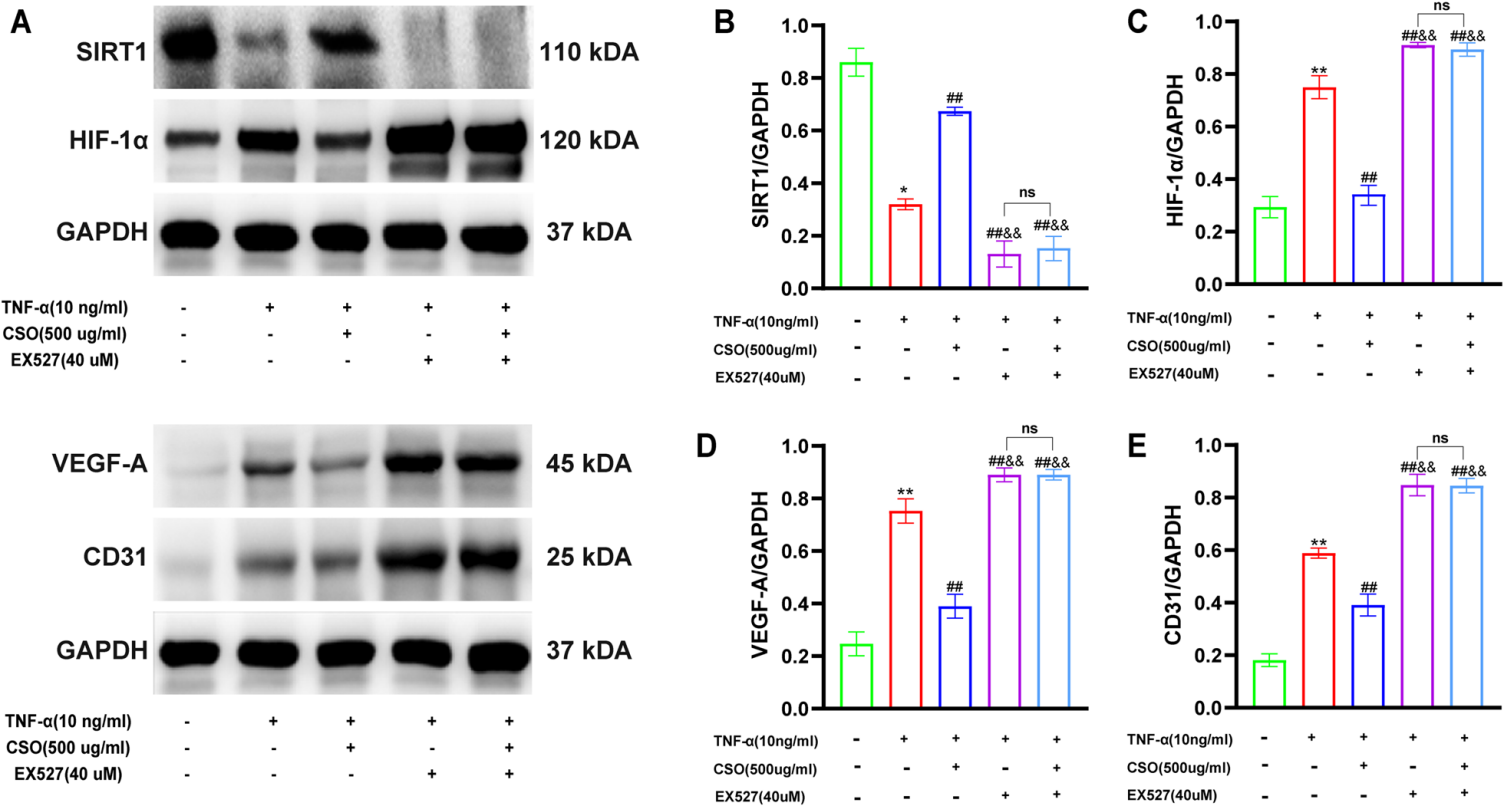
CSO modulated SIRT1, HIF-1α, VEGF-A, and CD31 expression in TNF-α induced FLS. **A** Representative picture of Western blot. **B** SIRT1/GAPDH. **C** HIF-1α/GAPDH. **D** VEGF-A/GAPDH. **E** CD31/GAPDH. Datasets are shown as the mean ± SD. *P < 0.05, **P < 0.01 vs. NC group; ^#^*P* < 0.05, ^##^*P* < 0.01 vs. TNF-α group; ^&^*P* < 0.05, ^&&^*P* < 0.01 vs. TNF-α + CSO group, n = 3

As shown in Fig. 10, we investigated the impact of CSO on the protein levels of SIRT1, HIF-1α, VEGF-A, and CD31 in TNF-α induced FLS. The SIRT1 level in the TNF-α group was considerably lower than that in the NC group, while the levels of HIF-1α and VEGF-A levels significantly elevated. Upon CSO treatment, there was a substantial increase in SIRT1 level, accompanied by a noticeable decrease in HIF-1α and VEGF-A levels. To further elucidate the relationship among SIRT1, HIF-1α, and VEGF-A in TNF-α induced FLS, we introduced EX527 to suppress SIRT1 expression. This resulted in a marked increase in HIF-1α and VEGF-A levels, suggesting a negative regulatory role of SIRT1 on the expression of HIF-1α and VEGF-A. Moreover, no substantial variations in SIRT1, HIF-1α, and VEGF-A levels were observed between the TNF-α + EX527 and TNF-α + EX527 + CSO groups. However, the TNF-α + EX527 + CSO group significantly differed from the TNF-α + CSO group in these levels. These findings suggested that CSO suppressed HIF-1α and VEGF-A levels by regulating SIRT1 in TNF-α induced FLS. Similarly, CSO notably reduced the expression of CD31 in TNF-α induced FLS. However, the inhibition of SIRT1 expression alleviated the ability of CSO to downregulate CD31 expression.

### CSO inhibited the TNF-α induced nuclear translocation of HIF-1α in FLS

**Fig. 11:**
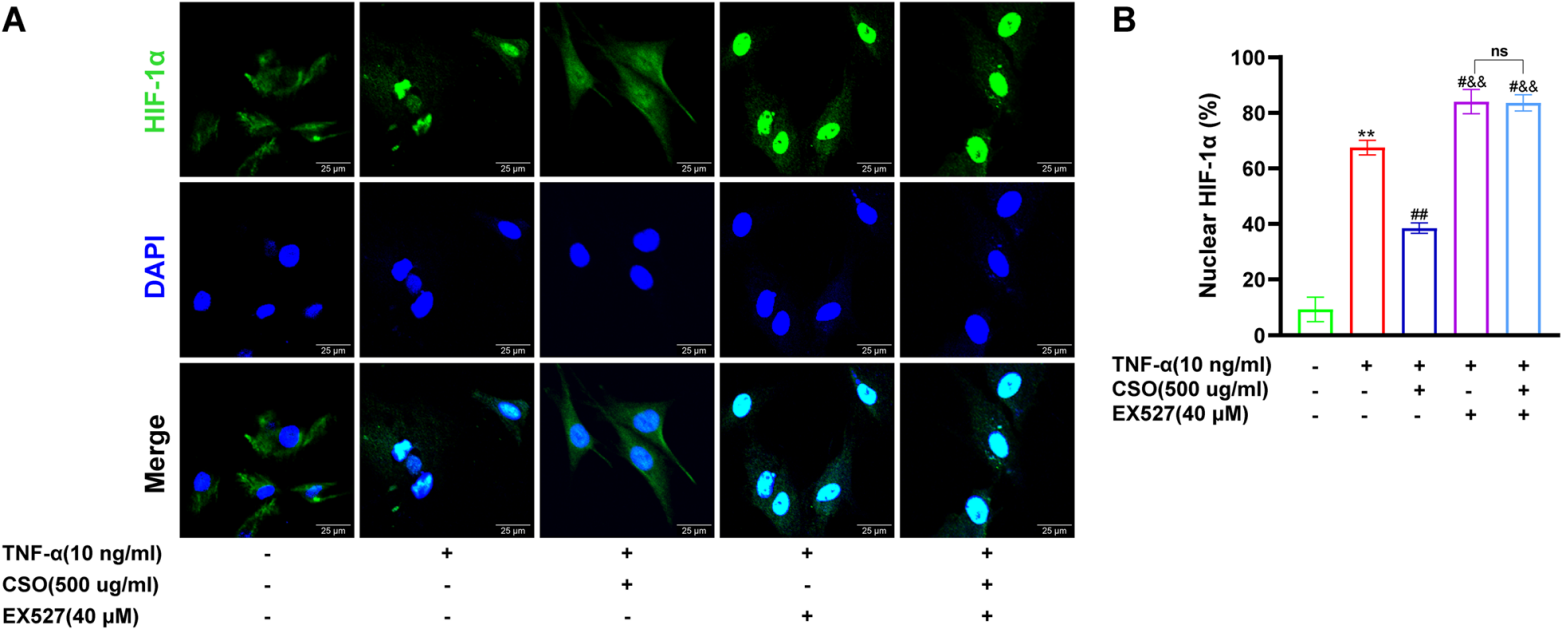
CSO inhibited TNF-α induced nuclear translocation of HIF-1α in TNF-α induced FLS. **A** Representative picture of HIF-1α translocation. **B** Nuclear HIF-1α rate. Datasets are shown as the mean ± SD. *P < 0.05, **P < 0.01 vs. NC group; ^#^*P* < 0.05, ^##^*P* < 0.01 vs. TNF-α group; ^&^*P* < 0.05, ^&&^*P* < 0.01 vs. TNF-α + CSO group, n = 3

We subsequently investigated the effect of CSO on HIF-1α nuclear translocation in FLS. As depicted in Fig. 11, there was minimal HIF-1α expression in the nucleus of FLS in the NC group. In the TNF-α group, a significant amount of HIF-1α was detected in the nucleus. However, this nuclear translocation of HIF-1α, which was notably prominent in the TNF-α group, was significantly diminished following CSO treatment. When SIRT1 expression was inhibited by EX527, the TNF-α + EX527 group showed a heightened nuclear accumulation of HIF-1α in comparison to the TNF-α group. However, it is noteworthy that the nuclear HIF-1α levels of the TNF-α + EX527 and TNF-α + EX527 + CSO groups were comparable. Furthermore, the nuclear HIF-1α levels were significantly different between the TNF-α + EX527 + CSO group and TNF-α + CSO group. These results implied that inhibiting SIRT1 expression in TNF-α induced FLS promoted HIF-1α nuclear translocation. Additionally, CSO could regulate the expression of SIRT1 to prevent HIF-1α nuclear translocation in TNF-α induced FLS.

### CSO inhibited angiogenesis of aortic rings

**Fig. 12:**
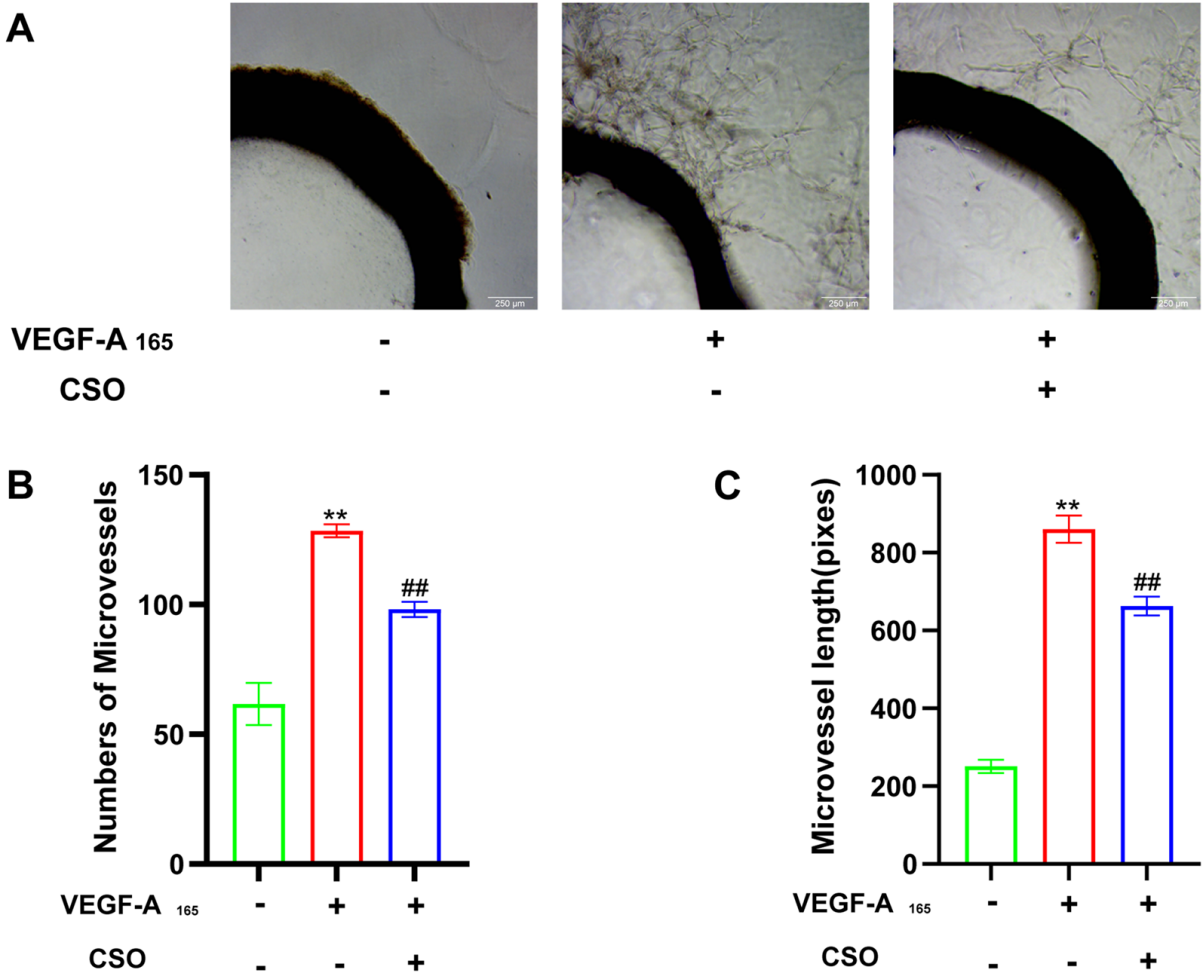
CSO inhibited the isolated aortic rings’ angiogenesis. **A** Representative picture of aortic rings angiogenesis. **B** Numbers of micro-vessels. **C** Micro-vessel length (pixels). Datasets are shown as the mean ± SD. *P < 0.05, **P < 0.01 vs. NC group; ^#^*P* < 0.05, ^##^*P* < 0.01 vs. VEGF-A_165_ group, n = 3

To further confirm the anti-angiogenesis effects of CSO, we conducted rat-isolated aortic ring angiogenesis experiments. As shown in Fig. 12, no micro-vessels formed around the aortic rings in the NC group. In contrast, the VEGF-A_165_ group exhibit a significantly higher number and length of micro-vessels compared to the NC group. Furthermore, CSO treatment markedly reduced the number and length of micro-vessels relative to the VEGF-A_165_ group. This additional evidence further supported the anti-angiogenesis effect of CSO ex vivo.

## Discussion

Angiogenesis is a tightly regulated process characterized by the sprouting of new blood vessels from pre-existing ones. The process involves vessel dilation, increased permeability, membrane degradation, migration of ECs, lumen formation, and recruitment of pericyte. It plays a crucial role in diverse physiological processes, such as wound healing, embryonic development, and menstrual cycle-related remodeling [30]. However, uncontrolled angiogenesis can have detrimental effects, including promoting tumor growth and contributing to vision loss in conditions like Age-Related Macular Degeneration and diabetic retinopathy [31]. In RA, angiogenesis is continuously stimulated throughout the disease progression, leading to persistent cartilage and bone destruction even during periods of clinical remission [32]. The CIA rat model is commonly employed to study the pathogenesis of RA as it exhibits comparable characteristics such as immune dysfunction and synovial angiogenesis [33]. In this study, the CIA rats exhibited increased arthritis index, visible paw swelling, increased immature vessels labeled with CD31^+^ /αSMA^−^. Moreover, we also observed pronounced inflammatory infiltration, extensive cartilage erosion, and joint destruction. Notably, the administration of high-dose CSO for four weeks demonstrated similar efficacy to MTX in treating synovial angiogenesis in vivo. These findings indicated that CSO exerted an anti-angiogenesis effect in CIA rats.

The mechanism of angiogenesis in RA primarily revolves around the dynamic, timely orchestrated, and spatially-coordinated interactions among FLS, ECs, and macrophages [34]. The RA synovial membrane consists mainly of FLS and macrophage-like synoviocytes. As key effector cells, FLS undergo abnormal activation and transformation, acquiring an aggressive phenotype that promotes arthritis progression. Inflammatory factors released by RA FLS, such as TNF-α, IL-8, and IL-6 can directly activate ECs or stimulate FLS to secrete growth factors like VEGF, indirectly promoting angiogenesis [35, 36]. VEGF-A, the predominant regulator of angiogenesis among VEGF isoforms (VEGF A-E), is elevated in the plasma and synovial fluid of RA patients [3]. Previous studies have shown that VEGF-A increases the functionality of ECs via the VEGFR2/PKC/ERK1/2 pathway [37]. Reducing VEGF-A expression improved pathological deterioration in CIA rats [4]. Consistent with prior studies, we found noticeable VEGF-A elevation in the serum and synovial tissue of CIA rats and TNF-α induced FLS. However, CSO treatment markedly decreased VEGF-A levels and inhibited micro-vessels formation around VEGF-A_165_ induced aortic rings. These findings suggested that CSO exerted an anti-angiogenesis effect by suppressing VEGF-A expression.

VEGF-A expression in the RA synovium is inextricably linked to hypoxia [38]. The RA synovium exhibits lower partial pressure of oxygen levels (18–33 mm Hg) compared to most normal organs (40–100 mm Hg) [39]. Increased FLS proliferation amplifies metabolic demands and creates a larger gap between FLS and adjacent blood vessels, resulting in insufficient oxygen supply. Additionally, periarticular stress caused by FLS proliferation, interstitial fluid outflow, and joint movements leads to vascular collapse and diminished blood flow, exacerbating hypoxia in the synovial tissue [8]. Hypoxia triggers the activation of the transcription factor HIF-1, which consists of HIF-1α and HIF-1β subunits. HIF-1α is controlled by oxygen levels, while HIF-1β maintains a constitutive phenotype in the nucleus. Under normoxic conditions, prolyl hydroxylase (PHD) hydroxylates the ODD structural domain of HIF-1α, leading to its subsequent degradation by E3 ubiquitin ligase [40]. PHD activity is inhibited under hypoxic conditions, allowing HIF-1α to translocate to the nucleus and form the activated HIF-1 heterodimer with HIF-1β. The activated complex binds to hypoxia response elements (HRE) on the promoter regions of target genes like VEGF-A. The HIF-1α/VEGF-A pathway establishes a positive feedback loop under hypoxic conditions, driving continuous angiogenesis [41]. Previous studies have shown increased HIF-1α expression in CIA rats during the first immunization, suggesting its potential involvement in early angiogenesis in arthritis [42]. Additionally, HIF-1α deficiency in CIA mice alleviated myeloid cells infiltration and disease progression [43]. In our study, CSO treatment strongly alleviated the increase in HIF-1α protein expression and inhibited HIF-1α nuclear translocation in CIA rats and TNF-α induced FLS. These results indicated that CSO had the potential to inhibit angiogenesis through the HIF-1α/VEGF-A pathway.

HIF-1α activation is not only triggered by hypoxia but also by various conditions such as temperature, growth regulators, and reactive oxygen species (ROS) [8]. Recent advancement in metabolomics techniques have revealed that dysfunctional energy metabolism in FLS is a fundamental characteristic of RA across all stages [44]. ROS generated from inflamed synovial tissue impairs FLS mitochondrial DNA, limits the activity of the electron transport chain (ETC) complex, and induces lipid peroxidation, leading to mitochondrial dysfunction that impedes NADH conversion to NAD+ [9]. SIRT1, an NAD+-dependent deacetylase, is downregulated in RA compared to osteoarthritis. Overexpression of SIRT1 has been shown to alleviate joint inflammation in RA [45]. Interestingly, SIRT1 has a dual effect on blood vessel formation, with SIRT1 deficiency inhibiting this process [46], and SIRT1 activation inhibiting RA angiogenesis through the MAPK and Rho/Rock pathway [11, 47]. Our study revealed significantly decreased levels of SIRT1 in CIA rats and TNF-α induced FLS, which were upregulated by CSO treatment. This suggested that CSO upregulated SIRT1 expression to exert anti-angiogenic effects. To further elucidate the mechanism of CSO’s anti-angiogenesis, we introduced the SIRT1 inhibitor EX527 to investigate the relationship between SIRT1 and HIF-1α and clarify whether SIRT1 is the direct target of CSO.

The correlation between SIRT1 and HIF-1α remains controversial [48, 49]. Some studies propose that SIRT1 inactivation diminishes HIF-1α activity by inhibiting p300 recruitment and suppressing HIF-1 target genes [50]. Another study suggests that SIRT1 overexpression enhances the stability of HIF-1α protein under hypoxic conditions [51]. In our study, inhibiting SIRT1 expression increased HIF-1α levels and nuclear translocation in TNF-α induced FLS. No significant differences were observed in migration, HIF-1α/VEGF-A protein levels, and HIF-1α nuclear translocation between TNF-α + EX527 + CSO and EX527 + CSO groups. Moreover, CSO treatment did not effectively reduce HIF-1α and VEGF-A protein levels when SIRT1 was inhibited, suggesting that CSO suppressed the HIF-1α / VEGF-A pathway by upregulating SIRT1.

Our findings indicate that CSO can alleviate angiogenesis in CIA rats. However, the study has some limitations due to the absence of experiments involving human umbilical vein endothelial cells (HUVECs). Given the critical role of VEGF-A as an activator of HUVECs in the angiogenesis process, the omission of HUVECs experiments may restrict our comprehensive understanding of the impact of CSO on RA angiogenesis. Future research should utilize a co-culture system of RA FLS and HUVECs to further explore the impact of CSO on angiogenesis.

## Conclusions

In summary, this study indicates demonstrated for the first time that CSO significantly inhibits synovial angiogenesis in CIA rats. Its underlying molecular mechanism may involve the suppression of HIF-1α/VEGF-A pathways by increasing SIRT1 expression in FLS. Therefore, these findings provide an important theoretical basis for the potential application in the treatment of RA.

### Supplementary Information

**Additional file 1: **Effect of CSO on viability of TNF-α induced FLS. A FLS viability (%), n = 6.

**Additional file 2: **Effect of CSO on apoptosis of FLS. A Representative FACS scatterplot of the Negative Control (NC) group. B Representative FACS scatterplot of the CSO (500 μg/ml) group. C Representative FACS scatterplot of the TNF-α (10 ng/ml) group. D Representative FACS scatterplot of the TNF-α (10 ng/ml) + CSO (500 μg/ml) group. E Percentage of apoptosis cells (%). Datasets are shown as the mean ± SD. *P<0.05, **P<0.01 vs. NC group. n=3.

## Data Availability

The datasets generated for this study are available from the corresponding author upon reasonable request.

## Abbreviations

RA: Rheumatoid arthritis
CSO: Coix seed oil
CIA: Collagen-induced arthritis
MTX: Methotrexate
FLS: Fibroblast-like synoviocytes
SIRT1: Silent information regulator of transcription 1
HIF-1α: Hypoxia inducible factor-1α
VEGF-A: Vascular endothelial growth factor-A
IC50: The half maximal inhibitory concentration
TNF-α: Tumor necrosis factor-α
WB: Western blot
IHC: Immunohistochemical
IF: Immunofluorescence
CCK8: Cell counting kit
