# Supplementary material for: Coix seed oil alleviates synovial angiogenesis through suppressing HIF-1α/VEGF-A signaling pathways via SIRT1 in collagen-induced arthritis rats
Source: Chin Med. 2023 Sep 15;18:119. doi: 10.1186/s13020-023-00833-6 (PMC10504826; doi:10.1186/s13020-023-00833-6)
Supplement: Supplementary file 1 — Additional file 1: Effect of CSO on viability of TNF-α induced FLS. A FLS viability (%), n = 6. [file 13020_2023_833_MOESM1_ESM.pptx]

## Slide 1
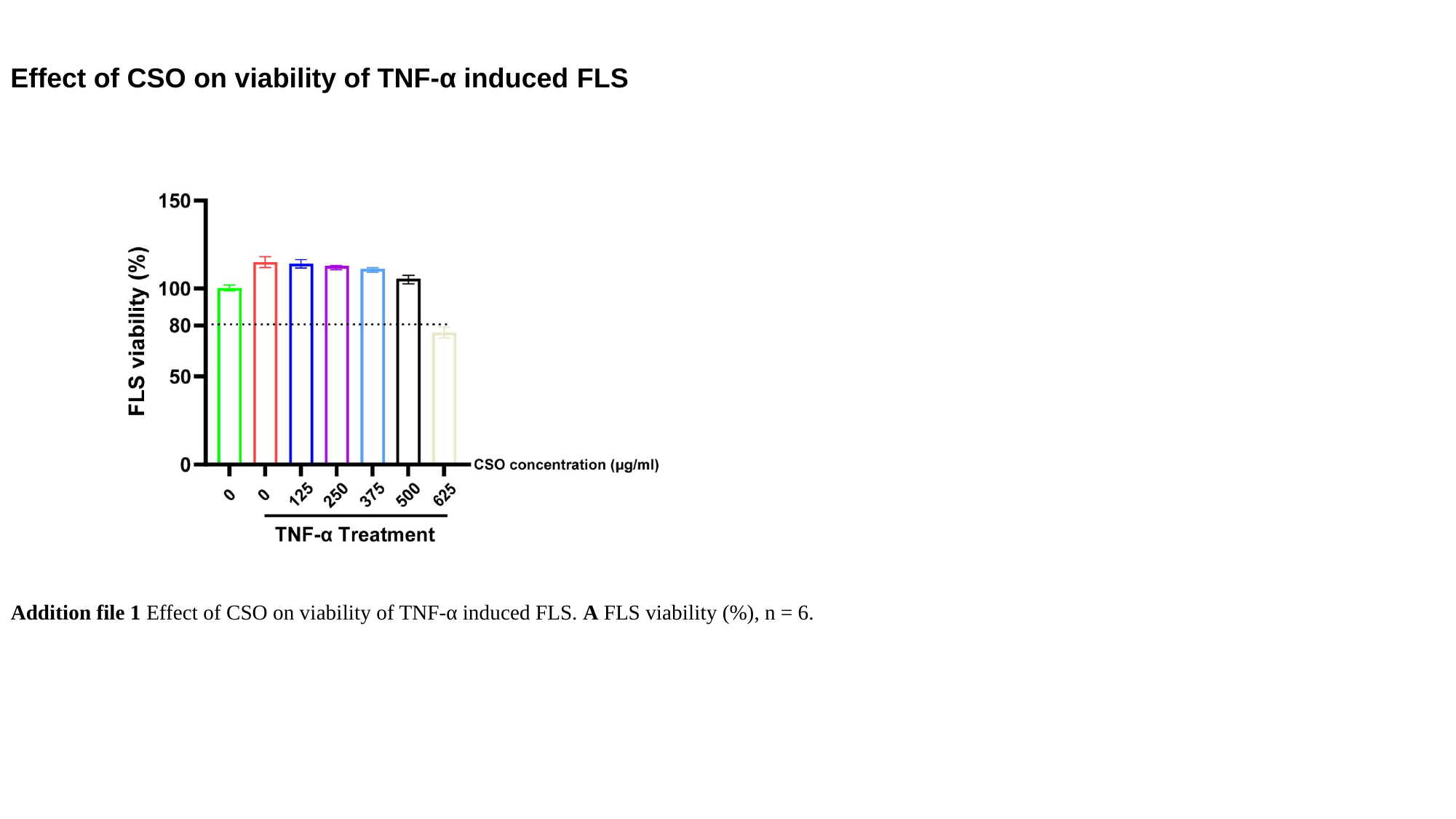

Effect of CSO on viability of TNF-α induced FLS
Addition file 1 Effect of CSO on viability of TNF-α induced FLS. A FLS viability (%), n = 6.
