# Supplementary material for: Coix seed oil alleviates synovial angiogenesis through suppressing HIF-1α/VEGF-A signaling pathways via SIRT1 in collagen-induced arthritis rats
Source: Chin Med. 2023 Sep 15;18:119. doi: 10.1186/s13020-023-00833-6 (PMC10504826; doi:10.1186/s13020-023-00833-6)
Supplement: Supplementary file 2 — Additional file 2: Effect of CSO on apoptosis of FLS. A Representative FACS scatterplot of the Negative Control (NC) group. B Representative FACS scatterplot of the CSO (500 μg/ml) group. C Representative FACS scatterplot of the TNF-α (10 ng/ml) group. D Representative FACS scatterplot of the TNF-α (10 ng/ml) + CSO (500 μg/ml) group. E Percentage of apoptosis cells (%). Datasets are shown as the mean ± SD. *P<0.05, **P<0.01 vs. NC group. n=3. [file 13020_2023_833_MOESM2_ESM.pptx]

## Slide 1
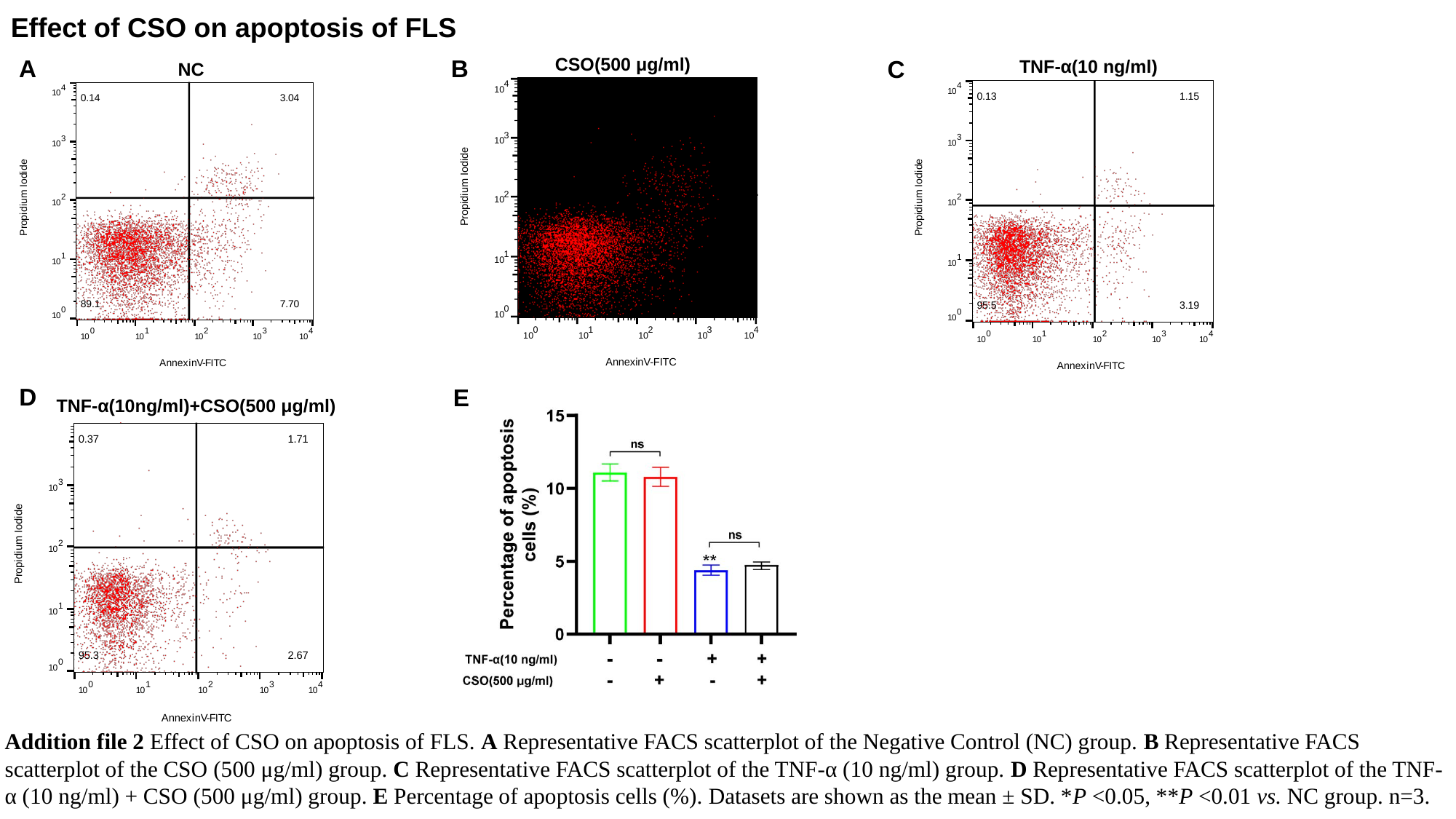

Effect of CSO on apoptosis of FLS
CSO(500 μg/ml)
A
B
4
10
0.16
2.51
3
10
Propidium Iodide
2
10
1
10
89.4
7.90
0
10
0
1
2
3
4
10
10
10
10
10
AnnexinV-FITC
C
TNF-α(10 ng/ml)
NC
D
E
TNF-α(10ng/ml)+CSO(500 μg/ml)
Addition file 2 Effect of CSO on apoptosis of FLS. A Representative FACS scatterplot of the Negative Control (NC) group. B Representative FACS scatterplot of the CSO (500 μg/ml) group. C Representative FACS scatterplot of the TNF-α (10 ng/ml) group. D Representative FACS scatterplot of the TNF-α (10 ng/ml) + CSO (500 μg/ml) group. E Percentage of apoptosis cells (%). Datasets are shown as the mean ± SD. *P <0.05, **P <0.01 vs. NC group. n=3.
